# Supplementary material for: Exploring Antioxidant Properties of Standardized Extracts from Medicinal Plants Approved by the Thai FDA for Dietary Supplementation
Source: Nutrients. 2025 Mar 4;17(5):898. doi: 10.3390/nu17050898 (PMC11901555; doi:10.3390/nu17050898)
Supplement: Supplementary file 1 [file nutrients-17-00898-s001.zip › nutrients-3432147-supplementary.pdf]

Supplementary Materials

Exploring Antioxidant Properties of Standardized Extracts from Medicinal Plants Approved by the Thai FDA for Dietary Supplementation

Surasak Limsuwan [1,†], Nurulhusna Awaeloh [2,†], Pinanong Na-Phatthalung [3], Thammarat Kaewmanee [4,*] and Sasitorn Chusri [2,*]

Table S1. The list of dietary supplements approved by the Thai Food and Drug Administration (FDA) derived from medicinal plants.

No	Scientific name	Common name	Family herbs	Part used	Domestically cultivated
1	*Actinidia arguta* (Siebold & Zucc.) Planch. ex Miq.	Arguta kiwifruit	Actinidiaceae	Fruit	X
2	*Actinidia chinensis* Planch.	Kiwifruit	Actinidiaceae	Fruit	√
3	*Aegle marmelos* (L.) Corrêa	Bael fruit	Rutaceae	Ripe fruit	√
4	*Allium cepa* L.	Onion	Amaryllidaceae	Rhizome	√
5	*Allium fistulosum* L.	Onion	Amaryllidaceae	Leaf/ Stem	√
6	*Allium sativum* L.	Garlic	Amaryllidaceae	Rhizome	√
7	*Allium schoenoprasum* L.	Chives	Amaryllidaceae	Rhizome	√
8	*Aloe vera* (L.) Burm.f.	Aloe	Asphodelaceae	Gel	√
9	*Alpinia galanga* (L.) Willd.	Galangal	Zingiberaceae	Rhizome	√
10	*Amorphophallus brevispathus* Gagnep.	NA	Araceae	Rhizome	√
11	*Ananas comosus* (L.) Merr.	Pineapple	Bromeliaceae	Fruit	√
12	*Angelica polymorpha* Maxim.	Dong quai	Apiaceae	Root	√
13	*Antidesma ghaesembilla* Gaertn.	Mamao	Phyllanthaceae	Fruit	√
14	*Apium graveolens* L.	Celery	Apiaceae	Leaf/ Stem	√
15	*Arctium lappa* L.	Burdock	Asteraceae	Root	√

Table S1. The list of dietary supplements approved by the Thai Food and Drug Administration (FDA) derived from medicinal plants.

No	Scientific name	Common name	Family	Part used	Domestically cultivated herbs
16	*Aristotelia chilensis* (Molina) Stuntz	Maqui berry	Elaeocarpaceae	Fruit	X
17	*Aronia melanocarpa* (Michx.) Elliott	Aronia berry	Rosaceae	Fruit	X
18	*Arthrospira maxima* Setchell & Gardner (AHN)	Spirulina	Phormidiaceae	Seaweed	X
19	*Arthrospira platensis* Gomont (AHN)	Spirulina	Phormidiaceae	Seaweed	X
20	*Ascophyllum nodosum*	Norwegian kelp	Fucaceae	Seaweed	X
21	*Asparagus officinalis* L.	Asparagus	Asparagaceae	Shoot/ Stem	√
22	*Astragalus propinquus* Schischkin	Astragalus/ Huang Qi	Fabaceae	Root	X
23	*Atractylodes macrocephala* Koidz.	White atractylodes	Asteraceae	Rhizome	X
24	*Auricularia auricular-* Judae	Ear of rock	Auriculariaceae	Mushroom	√
25	*Avena sativa* L.	Oat	Poaceae	Seed	X
26	*Bacopa monnieri* (L.) Wettst.	Brahmi	Plantaginaceae	Whole tree	√
27	*Beta vulgaris* L. var. *conditiva*	Beetroot	Amaranthaceae	Root	√
28	*Boesenbergia rotunda* (L.) Mansf.	Finger root	Zingiberaceae	Rhizome/ Root	√

Table S1. The list of dietary supplements approved by the Thai Food and Drug Administration (FDA), derived from medicinal plants.

No	Scientific name	Common name	Family herbs	Part used	Domestically cultivated
29	*Borago officinalis* L.	Borage	Boraginaceae	Seed	X
30	*Brassica oleracea* var. alboglabra (L.H.Bailey) Musil	Chinese broccoli	Brassicaceae	Leaf/ Stem	√
31	*Brassica oleracea italica x alboglabra*	Broccolini	Brassicaceae	Bloom/ Peduncle	X
32	*Brassica oleracea* L. var. *capitata* L.	Cabbage	Brassicaceae	Leaf/ Bloom	√
33	*Brassica oleracea* L. var. *italica* Plenck.	Broccoli	Brassicaceae	Bloom/ Stem/ Leaf	√
34	*Calendula officinalis* L.	Marigold	Asteraceae	Bloom	√
35	*Camellia sinensis* (L.) Kuntze	Green tea	Theaceae	Young shoots / Leaf/ Flower bud	√
36	*Canarium album* (Lour.) DC.	Chinese olive	Burseraceae	Fruit	√
37	*Capsicum annuum* L.	Chili	Solanaceae	Fruit	√
38	*Capsicum annuum* var.*grossum* (Willd.) Sendtn.	Peppers	Solanaceae	Fruit	√
39	*Capsicum frutescens* L.	Bird's eye chili	Solanaceae	Fruit	√
40	*Caralluma adscendens* var. fimbriata (Wall.) Gravely & Mayur.	NA	Apocynaceae	Ariel	X
41	*Carica papaya* L.	Papaya	Caricaceae	Ripe fruit	√

Table S1. The list of dietary supplements approved by the Thai Food and Drug Administration (FDA), derived from medicinal plants.

No	Scientific name	Common name	Family herbs	Part used	Domestically cultivated
42	*Carthamus tinctorius* L.	Safflower	Asteraceae	Bloom/ Seed	√
43	*Centella asiatica* (L.) Urb	Pennywort	Apiaceae	Leaf	√
44	*Chaenomeles speciosa* (Sweet) Nakai	Chinese quince	Rosaceae	Fruit	√
45	*Chlorella vulgaris* Beyerinck	Chlorella	Chlorellaceae	Seaweed	X
46	*Chrysanthemum indicum* L.	Chrysanthemum	Asteraceae	Bloom	√
47	*Chrysanthemum morifolium* Ramat.	Chrysanthemum	Asteraceae	Bloom	√
48	*Cichorium intybus* L.	Chicory	Asteraceae	Root	X
49	*Cinnamomum cassia* (L.) J. Presl	Cassia	Luaraceae	Bark	√
50	*Cinnamomum verum* J.Presl.	Cinnamon	Luaraceae	Bark	√
51	*Citrus aurantifolia* (Christm.) Swingle	Lime	Rutaceae	Fruit	√
52	*Citrus aurantium* L.	Sour orange	Rutaceae	Fruit	√
53	*Citrus limon* (L.) Burm.f.	Lemon	Rutaceae	Fruit	√
54	*Citrus paradise* Macfad	Grapefruit	Rutaceae	Fruit	X
55	*Citrus reticulate* Blanco	Mandarin	Rutaceae	Fruit	√

Table S1. The list of dietary supplements approved by the Thai Food and Drug Administration (FDA), derived from medicinal plants.

No	Scientific name	Common name	Family herbs	Part used	Domestically cultivated
56	*Citrus sinensis* (L.) Osbeck	Orange sweet	Rutaceae	Fruit	√
57	*Cladosiphon okamuranus*	Mozuku	Chordariaceae	Seaweed	X
58	*Cocos nucifera* L.	Coconut	Arecaceae	Fruit	√
59	*Codonopsis pilosula* (France) Nannf.	Radi Codonopsis	Campanulaceae	Root	X
60	*Coffea arabica* L.	Green coffee beans	Rubiaceae	Beans	√
61	*Coix lacryma-jobi* L.	Job's tears	Poaceae	Seed	√
62	*Cordyceps sinensis* (Berk.) Sacc.	Cordyceps	Clavicipitaceae	Whole tree	X
63	*Crataegus laevigata* (Poir.) DC.	Hawthorn	Rosaceae	Fruit	X
64	*Crataegus pinnatifida* Bunge	Hawthorn	Rosaceae	Fruit	X
65	*Cucumis melo* L.	Melon	Cucurbitaceae	Fruit	√
66	*Cucumis sativus* L.	Cucumber	Cucurbitaceae	Fruit	√
67	*Cucurbita pepo* supsp. *pepo*	Pumpkin	Cucurbitaceae	Fruit/ Seed	√
68	*Curcuma longa* L.	Tumeric	Zingiberaceae	Rhizome	√
69	*Cymbopogon citratus* (DC.) stapf	Lemongrass	Poaceae	Stem	√
70	*Cynara scolymus* L.	Artichoke	Asteraceae	Leaf	√

Table S1. The list of dietary supplements approved by the Thai Food and Drug Administration (FDA), derived from medicinal plants.

No	Scientific name	Common name	Family herbs	Part used	Domestically cultivated
71	*Daucus carota* L.	Carrot	Apiaceae	Root	√
72	*Dimocarpus longan* Lour.	Longan	Sapindaceae	Fruit	√
73	*Dioscorea polystachya* Turcz.	Chinese yam	Dioscoreaceae	Rhizome	X
74	*Dunaliella salina*	D salina	Dunaliellaceae	Seaweed	X
75	*Eleutherococcus senticosus* (Rupr. & Maxim.) Maxim.	Siberian ginseng	Araliaceae	Root	X
76	*Equisetum arvense* L.	Horsetail	Equisetaceae	Stem	X
77	*Equisetum hyemale* L.	Horsetail	Equisetaceae	Stem	X
78	*Eucommia ulmoides* Oliv.	Du Zhong/ Hardy rubber tree	Eucommiaceae	Bark	X
79	*Euterpe oleracea* Mart.	Acai	Arecaceae	Fruit	X
80	*Fagopyrum esculentum* Moench	Buckwheat	Polygonaceae	Seed	X
81	*Fagopyrum tataricum* (L.) Gaertn	Tartary Buckwheat	Polygonaceae	Seed	X
82	*Foeniculum vulgare* Mill.	Fennel	Apiaceae	Seed	X
83	*Fragaria* x *ananassa* (Weston) Duchesne	Strawberry	Rosaceae	Fruit	√
84	*Ganoderma lucidum* (Curtis) P. Karst.	Reishi mushroom	Ganodermata-ceae	Mushroom	√

Table S1. The list of dietary supplements approved by the Thai Food and Drug Administration (FDA), derived from medicinal plants.

No	Scientific name	Common name	Family	Part used	Domestically cultivated herbs
85	*Garcinia atroviridis* Griff. ex T.Anderson	Asam gelugor	Clusiaceae	Fruit	√
86	*Garcinia gummi-gutta* (L.) Roxb.	Gamboge	Clusiaceae	Fruit	√
87	*Garcinia mangostana* L.	Mangosteen	Clusiaceae	Fruit	√
88	*Ginkgo biloba* L.	Ginkgo	Ginkgoaceae	Leaf	√
89	*Glycine max* (L.) Merr.	Soybean	Fabaceae	Seed	√
90	*Glycyrrhiza glabra* L.	Liquorice	Fabaceae	Root	√
91	*Glycyrrhiza uralensis* Fisch.	Chinese Liquorice	Fabaceae	Root	√
92	*Grifola frondosa* (Dicks.) Gray	Maitake	Meripilaceae	Mushroom	√
93	*Gynostemma pentaphyllum* (Thunb.) Makino	Jiao Gu Lan	Cucubitaceae	Leaf/ Stem	√
94	*Haematococcus pluvialis*	NA	Hematococcaceae	Seaweed	X
95	*Helianthus annuus* L.	Sunflower	Asteraceae	Seed	√
96	*Helianthus tuberosus* L.	Jerusalem artichoke	Asteraceae	Rhizome	√
97	*Hericum erinaceus* (Bull.) Pers	Lion’s mane mushroom	Hericiaceae	Mushroom	√
98	*Hibiscus sabdariffa* L.	Roselle	Malvaceae	Sepal / Bloom	√
99	*Hippophae rhamnoides* L.	Sea buckthorn	Elaeagnaceae	Fruit	√

Table S1. The list of dietary supplements approved by the Thai Food and Drug Administration (FDA) derived from medicinal plants.

No	Scientific name	Common name	Family	Part used	Domestically cultivated herbs
100	*Hordeum vulgare* L.	Barley	Poaceae	Young leaves/ Seed/ Endosperm	√
101	*Houttuynia cordata* Thunb.	Plu kaow	Saururaceae	Leaf	√
102	*Illicium verum* Hook.f.	Star anise/ Chinese anise	Schisandraceae	Fruit	√
103	*Ipomoea aquatica* Forssk.	Water spinach	Convolvulaceae	Stem/ Leaf	√
104	*Kaempferia parviflora* wall.Ex Baker	Black galingale	Zingiberaceae	Rhizome	√
105	*Laminaria digitate*	Kelp/ Brown algae	Laminariaceae	Seaweed	X
106	*Laminaria japonica*	Japanese kelp/ Kombu/ Haidai	Laminariaceae	Seaweed	X
107	*Laminaria longicruris*	NA	Laminariaceae	Seaweed	X
108	*Laminaria sinclairii*	NA	Laminariaceae	Seaweed	X
109	*Lentinula edodes* (Berk.) Pegler	Shiitake	Marasmiaceae	Mushroom	√
110	*Linum usitatissimum* L.	Linseed/ Flaxseed	Linaceae	Seed	√
111	*Litchi chinensis* Sonn.	Litchi	Sapindaceae	Ariel	√
112	*Lithothamnion calcareum*	Red marine algae	Hapalidiaceae	-	X

Table S1. The list of dietary supplements approved by the Thai Food and Drug Administration (FDA), derived from medicinal plants.

No	Scientific name	Common name	Family herbs	Part used	Domestically cultivated
113	*Lonicera japonica* Thunb.	Japanese honeysuckle	Caprifoliaceae	Bloom	√
114	*Lycium barbarum* L.	Goji berry	Solanaceae	Fruit	√
115	*Lycopersicon esculentum* Mill.	Tomato	Solanaceae	Fruit	√
116	*Malpighia glaba* L.	Barbadoscherry/ Acerola cherry	Malpighiaceae	Fruit	√
117	*Malus domestica* Borkh.	Apple	Rosaceae	Fruit	√
118	*Mangifera indica* L.	Mango	Anacardiaceae	Fruit	√
119	*Matricaria chamomilla* L.	Chamomile	Asteraceae	Bloom	√
120	*Medicago sativa* L.	Alfalfa	Fabaceae	Leaf/ Stem	√
121	*Melissa officinalis* L.	Balm	Lamiaceae	Leaf	√
122	*Mentha* x *piperita* L.	Peppermint	Lamiaceae	Stem/ Leaf	√
123	*Mentha spicata* L.	Spearmint	Lamiaceae	Leaf	√
124	*Momordica Cochinchinensis* (Lour.) Spreng.	Gac/ Spiny Bitter gourd	Cucurbitaceae	Fruit/ Seed coat	√
125	*Momordica grosvenorii* Swingle	Luo han guo	Cucurbitaceae	Fruit	√
126	*Morinda citrifolia* L.	Noni	Rubiaceae	Fruit/ Leaf	√
127	*Moringa oleifera* Lam.	Ben moringa/ Drumstick tree	Moringaceae	Young leaves	√
128	*Morus alba* L.	Mulberry	Moraceae	Leaf/ Fruit	√

Table S1. The list of dietary supplements approved by the Thai Food and Drug Administration (FDA), derived from medicinal plants.

No	Scientific name	Common name	Family	Part used	Domestically cultivated herbs
129	*Musa sapientum* L.	Banana	Musaceae	Ripe fruit	√
130	*Myrciaria dubia* (Kunth) McVaugh	Rumberry/ Camu-camu	Myrtaceae	Fruit	√
131	*Ocimum basilicum* L.	Basil/ Sweet basil	Lamiaceae	Leaf/ Bloom	√
132	*Oenothera biennis* L.	Evening primrose	Onagraceae	Seed	√
133	*Olea europaea* L.	Table olive	Oleaceae	Fruit	√
134	*Ophiopogon japonicas* (Thunb.) Ker Gawl.	Lily turf	Asparagaceae	Root/ Fruit	X
135	*Opuntia ficus-indica* (L.) Mill.	Prickly pear	Cactaceae	Fruit/ Leaf/ Stem	√
136	*Opuntia robusta* J.C. Wendl.	NA	Cactaceae	Stem	√
137	*Origanum vulgare* L.	Oregano	Lamiaceae	Leaf	√
138	*Oryza sativa* L.	Rice	Poaceae	Seed/ Germ	√
139	*Paeonia lactiflora* Pall.	Chinese peony	Paeoniaceae	Root	√
140	*Palmaria palmata* L.	Dulse	Palmariaceae	Seaweed	X
141	*Panax ginseng* C.A. Mey.	Korea ginseng	Araliaceae	Root	X
142	*Panax notoginseng* (Burkill) F.H.Chen	Sanchi root	Araliaceae	Root	X

Table S1. The list of dietary supplements approved by the Thai Food and Drug Administration (FDA), derived from medicinal plants.

No	Scientific name	Common name	Family	Part used	Domestically cultivated herbs
143	*Panax quinquefolius* L.	American ginseng	Araliaceae	Root	X
144	*Panicum miliaceum* L.	Millet	Poaceae	Seed	√
145	*Passiflora edulis* Sims.	Passion fruit	Passifloraceae	Fruit	√
146	*Perilla frutescens* (L.) Britton	Perilla	Lamiaceae	Seed	√
147	*Persea americana* Mill.	Avocado	Luaraceae	Fruit	√
148	*Petroselinum crispum* (Mill.) Fuss	Parsley	Apiaceae	Aril/ Leaf/ Seed Seed	√
149	*Phaseolus mungo* L.	Urd bean	Fabaceae	Seed	√
150	*Phaseolus vulgaris* L.	Bean	Fabaceae	Seed	√
151	*Phyllanthus emblica* L.	Indian gooseberry	Phyllanthaceae	Fruit	√
152	*Pinus massoniana* Lamb.	Pine	Pinaceae	Fruit	√
153	*Pinus pinaster* Aiton	Frenc maritime pine	Pinaceae	Bark	X
154	*Pinus strobus* L.	Pine bark	Pinaceae	Bark	X
155	*Piper nigrum* L.	Paper/ Black pepper	Piperaceae	Seed	√
156	*Piper sarmentosum* Roxb.	Wild betle leaf bush	Piperaceae	Leaf	√
157	*Pisum sativum* L.	Pea	Fabaceae	Pod/ Seed	√
158	*Plantago ovate* Forssk.	Psyllium seed	Plantaginaceae	Seed/ Seed coat	√

Table S1. The list of dietary supplements approved by the Thai Food and Drug Administration (FDA), derived from medicinal plants.

No	Scientific name	Common name	Family	Part used	Domestically cultivated herbs
159	*Pleurotus ostreatus* (Jacq.) P. Kumm	Oyste mushroom	Pleurotaceae	Mushroom	√
160	*Plukenetia volubilis* L.	Sacha inchi/ Incapeanut	Euphobiaceae	Seed	√
161	*Polygonatum odoratum* (Mill.) Druce	Polygonatum	Asparagaceae	Rhizome	X
162	*Poria cocos* (Schw.) Wolf	Indian bread	Polyporaceae	Sclerotium / Root	X
163	*Prunus armeniaca* L.	Apricot	Rosaceae	Fruit	√
164	*Prunus cerasus* L.	Sour cherry/ Tart cherry/ Sweet cherry	Rosaceae	Fruit	√
165	*Prunus domestica* L.	Plum	Rosaceae	Fruit	√
166	*Prunus mume* (Siebold) Siebold & Zucc.	Japanese apricot	Rosaceae	Fruit	√
167	*Psidium guajava* L.	Guava	Myrtaceae	Fruit	√
168	*Pueraria lobate* (Willd.) Ohwi	Kudzu	Fabaceae	Root	X
169	*Punica granatum* L.	Pomegranate	Punicaceae	Seed/ Aril	√
170	*Pyrus arbustifolia* (L.) Pers	Aronia	Rosaceae	Fruit	X
171	*Rehmannia glutinosa* (Gaertn.) DC.	Rehmannia	Scrophularia-ceae	Root	X

Table S1. The list of dietary supplements approved by the Thai Food and Drug Administration (FDA), derived from medicinal plants.

No	Scientific name	Common name	Family	Part used	Domestically cultivated herbs
172	*Ribes nigrum* L.	Blackcurrant/ Redcurrant	Grossulariaceae	Fruit/ Seed	X
173	*Rosa centifolia* L.	Rose	Rosaceae	Petal	√
174	*Rosa canina* L.	Rose hips	Rosaceae	Fruit	√
175	*Rosa spp.* and hybrid	Rose	Rosaceae	Petal	√
176	*Rosmarinus officinalis* L.	Rosemary	Lamiaceae	Leaf/ Branch	√
177	*Rubus fruticosus* G.N. Jones	Blackberry	Rosaceae	Fruit	√
178	*Rubus allegheniensis* Porter	Blackberry	Rosaceae	Fruit	X
179	*Rubus idaeus* L.	Red raspberry	Rosaceae	Fruit	√
180	*Rubus ursinus* cv. 'Young'	Youngberry	Rosaceae	Fruit	X
181	*Saccharum officinarum* L.	Sugar cane	Poaceae	Stem	√
182	*Salvia officinalis* L.	Sage	Lamiaceae	Leaf	√
183	*Salvia hispanica* L.	Chia	Labiaceae	Seed	√
184	*Sambucus canadensis* L.	Elderberry	Adoxaceae	Fruit	X
185	*Sambucus nigra* L.	Elderberry	Adoxaceae	Fruit	X
186	*Schisandra chinensis* (Turcz.) Baill.	Schisandraberry	Schisandraceae	Fruit	X
187	*Sesamum indicum* L.	Sesame	Pedaliaceae	Seed	√
188	*Solanum torvum* Sw.	Pea eggplant/ Plate brush	Solanaceae	Fruit	√

Table S1. The list of dietary supplements approved by the Thai Food and Drug Administration (FDA), derived from medicinal plants.

No	Scientific name	Common name	Family	Part used	Domestically cultivated herbs
189	*Spinacia oleracea* L.	Spinach	Amarathaceae	Leaf	√
190	*Syzygium cumini* (L.) Skeels	Black plum/ Jambolan	Myrtaceae	Fruit	√
191	*Tagetes erecta* L.	Marigold	Asteraceae	Bloom	√
192	*Tamarindus indica* L.	Tamarind	Fabaceae	Fruit	√
193	*Taraxacum dens leonis* Desr.	Dandelion	Asteraceae	Root	√
194	*Taraxacum mongolicum* Hard.Maz	Dandelion	Asteraceae	Root	√
195	*Terminalia bellirica* (Gaertn.) Roxb.	Belliric Myrobaran	Combretaceae	Fruit	√
196	*Terminalia chebula* Retz.	Chebulic myrobalan	Combretaceae	Fruit	√
197	*Thymus vulgaris* L.	Thyme	Lamiaceae	Leaf	√
198	*Tremella fuciformis* Berk	White jelly mushroom	Tremellaceae	Mushroom	√
199	*Trigonella-foenum graecum* L.	Fenugreek	Fabaceae	Seed	√
200	*Triticum aestivum* L.	Wheat	Poaceae	Seed	√
201	*Turnera diffusa* Willd. ex Schult.	Damiana	Passifloraceae	Leaf	X
202	*Undaria pinnatifida*	Wakame	Alariaceae	Seaweed	X
203	*Vaccinium angustifolium* Aiton	Blueberry	Ericaceae	Fruit	√

Table S1. The list of dietary supplements approved by the Thai Food and Drug Administration (FDA), derived from medicinal plants.

No	Scientific name	Common name	Family herbs	Part used	Domestically cultivated
204	*Vaccinium myrtillus* L.	Bilberry	Ericaceae	Fruit	X
205	*Vaccinium macrocarpon* Aiton	Cranberry	Ericaceae	Fruit	√
206	*Vaccinium uliginosum* L.	Bog bilberry	Ericaceae	Fruit	X
207	*Vitis labrusca* L.	Bordo grape	Vitaceae	Fruit/ Seed	√
208	*Vitis vinifera* L.	Grape	Vitaceae	Fruit/ Seed/ Bark	√
209	*Vigna radiata* (L.) R. Wilczek	Mungbean	Fabaceae	Seed	√
210	*Zingiber officinale* Rosc.	Ginger	Zingiberaceae	Rhizome	√
211	*Ziziphus jujuba* Mill.	Jujube/ Chinese jujube	Rhamnaceae	Fruit	√

*NA = Not available

Table S2 Antioxidant capacities of medicinal plants chosen from the list of Thai FDA-approved dietary supplements derived from medicinal plants.

No	Scientific name	Antioxidant capacities (mg/mL)		References
		DPPH	ABTS	
1	*Actinidia chinensis*	0.20	0.02	[1,2]
2	*Aegle marmelos*	0.06	0.02	[3]
3	*Allium cepa*	161.29	0.67	[4, 5]
4	*Allium fistulosum*	0.01	0.05	[6, 7]
5	*Allium sativum*	0.20	0.44	[8]
6	*Aloe vera*	0.003	0.003	[9, 10]
7	*Alpinia galanga*	10.66	0.09	[11, 12]
8	*Ananas comosus*	0.0001	0.0500	[13, 14]
9	*Angelica polymorpha*	0.01	0.10	[15, 16]
10	*Antidesma ghaesembilla*	0.11	0.02	[17]
11	*Apium graveolens*	1.09	0.71	[18]
12	*Bacopa monnieri*	0.79	1.11	[19]
13	*Beta vulgaris*	1.38	0.66	[20]
14	*Boesenbergia rotunda*	0.74	0.06	[21]
15	*Brassica oleracea*	0.74	16.95	[22, 23]
16	*Brassica oleracea*	15.71	17.04	[24]
17	*Brassica oleracea*	3.72	0.04	[25, 26]
18	*Calendula officinalis*	0.10	0.01	[27]
19	*Camellia sinensis*	0.010	0.006	[28]
20	*Capsicum frutescens*	0.004	0.070	[29]

Table S2 Antioxidant capacities of medicinal plants chosen from the list of Thai FDA-approved dietary supplements derived from medicinal plants.

No	Scientific name	Antioxidant capacities (mg/mL)		References
		DPPH	ABTS	
21	*Capsicum annuum* (peppers)	0.03	0.04	[30]
22	*Capsicum annuum* (bird eye's chilli)	0.020	0.003	[30]
23	*Carthamus tinctorius*	0.07	0.08	[31]
24	*Centella asiatica*	0.05	0.03	[32]
25	*Chaenomeles speciosa*	0.22	16.03	[33, 34]
26	*Chrysanthemum indicum*	2.21	0.20	[35, 36]
27	*Chrysanthemum morifolium*	1.69	2.13	[37]
28	*Cinnamomum cassia*	0.040	0.005	[38]
29	*Cinnamomum verum*	0.01	0.01	[39]
30	*Citrus aurantifolia*	2.36	0.26	[40]
31	*Citrus aurantium*	0.81	0.49	[41]
32	*Citrus limon*	0.03	0.03	[42]
33	*Citrus reticulate*	0.72	1.26	[43]
34	*Citrus sinensis*	29.70	4.17	[44]
35	*Cocos nucifera*	0.29	0.01	[45]
36	*Coffea arabica*	0.16	0.36	[46]
37	*Coix lacryma-jobi*	17.56	21.20	[47, 48]
38	*Cucumis melo*	0.02	17.56	[49, 50]
39	*Cucurbita pepo*	0.03	0.01	[51]
40	*Curcuma longa*	0.001	0.020	[52, 53]
41	*Cymbopogon citratus*	0.03	0.17	[54]

Table S2 Antioxidant capacities of medicinal plants chosen from the list of Thai FDA-approved dietary supplements derived from medicinal plants.

No	Scientific name	Antioxidant capacities (mg/mL)		References
		DPPH	ABTS	
42	*Cynara scolymus*	0.910	0.007	[55, 56]
43	*Daucus carota*	1.36	1.58	[57]
44	*Fragaria ananassa*	0.0004	0.0400	[58]
45	*Ganoderma lucidum*	0.050	0.001	[59]
46	*Garcinia atroviridis*	0.630	0.006	[60]
47	*Garcinia mangostana*	0.07	0.09	[61]
48	*Ginkgo biloba*	0.002	0.130	[62, 63]
49	*Glycine max*	0.11	0.08	[64]
50	*Glycyrrhiza uralensis*	0.05	0.05	[65]
51	*Gynostemma pentaphyllum*	0.06	0.07	[66]
52	*Helianthus annuus*	0.09	0.08	[67, 68]
53	*Helianthus tuberosus*	0.16	0.10	[69]
54	*Hibiscus sabdariffa*	0.01	0.02	[70]
55	*Hippophae rhamnoides*	0.1200	0.0008	[71]
56	*Hordeum vulgare*	0.04	0.01	[72]
57	*Houttuynia cordata*	0.10	0.42	[73]
58	*Illicium verum*	0.0005	0.0007	[74]
59	*Ipomoea aquatica*	0.39	0.39	[75]
60	*Kaempferia parviflora*	1.14	1.46	[76]
61	*Lentinula edodes*	0.05	0.02	[77]
62	*Linum usitatissimum*	0.05	0.05	[78]

Table S2 Antioxidant capacities of medicinal plants chosen from the list of Thai FDA-approved dietary supplements derived from medicinal plants.

No	Scientific name	Antioxidant capacities (mg/mL)		References
		DPPH	ABTS	
63	*Lonicera japonica*	0.01	0.02	[79]
64	*Lycium barbarum*	1.29	0.39	[80]
65	*Lycopersicon esculentum*	0.21	0.05	[81]
66	*Malus domestica*	0.0004	0.0002	[82]
67	*Matricaria chamomilla*	0.001	0.001	[83]
68	*Medicago sativa*	0.10	0.01	[84]
69	*Melissa officinalis*	0.010	0.001	[85]
70	*Mentha* x *piperita*	0.01	0.15	[86]
71	*Mentha spicata*	0.09	0.17	[86]
72	*Momordica cochinchinensis*	143.50	0.03	[87, 88]
73	*Momordica grosvenorii*	0.11	1.47	[89]
74	*Moringa oleifera*	1.87	1.36	[90]
75	*Morus alba*	6.65	1.40	[91]
76	*Musa sapientum*	0.050	0.002	[92, 93]
77	*Myrciaria dubia*	0.05	0.02	[94]
78	*Ocimum basilicum*	0.59	0.73	[95]
79	*Opuntia ficus-indica*	3.52	0.80	[96]
80	*Origanum vulgare*	0.10	0.09	[97]
81	*Oryza sativa*	1.09	2.80	[98]
82	*Paeonia lactiflora*	0.61	0.34	[99]
83	*Panicum miliaceum*	0.21	0.69	[100]

Table S2 Antioxidant capacities of medicinal plants chosen from the list of Thai FDA-approved dietary supplements derived from medicinal plants.

No	Scientific name	Antioxidant capacities (mg/mL)		References
		DPPH	ABTS	
84	*Persea americana*	0.010	0.001	[101]
85	*Petroselinum crispum*	281.02	587.98	[102]
86	*Phaseolus mungo*	488.00	1.12	[103, 104]
87	*Phaseolus vulgaris*	0.002	0.010	[105]
88	*Phyllanthus emblica*	0.05	0.30	[106]
89	*Pinus massoniana*	0.05	0.06	[107]
90	*Piper nigrum*	0.20	0.22	[108]
91	*Piper sarmentosum*	0.26	0.18	[109]
92	*Pisum sativum*	0.65	1.90	[110]
93	*Pleurotus ostreatus*	0.04	0.01	[111]
94	*Plukenetia volubilis*	0.04	0.04	[112]
95	*Prunus armeniaca*	83.86	0.47	[113]
96	*Prunus cerasus*	10.96	0.31	[114]
97	*Prunus domestica*	0.0900	0.0005	[113]
98	*Prunus mume*	0.005	0.020	[115]
99	*Punica granatum*	0.45	72.73	[116]
100	*Rosa centifolia*	0.92	0.07	[117]
101	*Rosmarinus officinalis*	0.10	0.18	[118]
102	*Rubus fruticosus*	3.53	0.50	[119]
103	*Rubus idaeus*	0.040	0.004	[120]
104	*Saccharum officinarum*	19.82	3.50	[121]
105	*Salvia hispanica*	0.001	0.001	[122]

Table S2 Antioxidant capacities of medicinal plants chosen from the list of Thai FDA-approved dietary supplements derived from medicinal plants.

No	Scientific name	Antioxidant capacities (mg/mL)		References
		DPPH	ABTS	
106	*Sesamum indicum*	8.88	24.91	[123]
107	*Solanum torvum*	0.02	0.01	[124]
108	*Tagetes erecta*	0.06	0.07	[125]
109	*Terminalia chebula*	0.002	0.003	[126]
110	*Thymus vulgaris*	0.01	0.05	[127]
111	*Trigonella-foenum graecum*	0.35	0.10	[128]
112	*Triticum aestivum*	0.05	0.01	[129]
113	*Vaccinium macrocarpon*	0.09	0.10	[130]
114	*Vitis vinifera*	0.27	0.04	[131]
115	*Vigna radiata*	0.07	0.08	[132]
116	*Zingiber officinale*	0.008	0.007	[133]

Table S3 Acetylcholinesterase inhibitory effects of medicinal plants chosen from the list of Thai FDA-approved dietary supplements derived from medicinal plants.

No	Scientific name	Acetylcholinesterase (AChE)*		References
		IC_{50} (mg/mL)	%inhibition (tested conc.)	
1	*Actinidia chinensis*	ND	79.8 (1 mg/mL)	[134]
2	*Aegle marmelos*	44.65	ND	[135]
3	*Allium cepa*	0.07	ND	[136]
4	*Allium fistulosum*	ND	21.31 (1 mg/mL)	[137]
5	*Allium sativum*	3.02	ND	[135]
6	*Aloe vera*	0.05	89.82 (0.1 M)	[138]
7	*Alpinia galanga*	0.03	ND	[139]
8	*Ananas comosus*	ND	9.30 (1 mg/mL)	[140]
9	*Angelica polymorpha*	ND	68.58 (50 µL)	[141]
10	*Antidesma ghaesembill*	ND	ND	
11	*Apium graveolens*	ND	4.70 (10 µL)	[142]
12	*Bacopa monnieri*	0.52	15.15 (1 mg/mL)	[143]
13	*Beta vulgaris*	0.0012	92.90 (100 µM)	[144]
14	*Boesenbergia rotunda*	ND	70.10 (20 µL)	[145]
15	*Brassica oleracea*	ND	ND	
16	*Brassica oleracea*	ND	ND	
17	*Brassica oleracea*	ND	ND	
18	*Calendula officinalis*	ND	5.23 (500 µg/mL)	[146]
19	*Camellia sinensis*	ND	81.66 (2 mg/mL)	[147]
20	*Capsicum frutescens*	0.08	ND	[148]
21	*Capsicum annuum* (peppers)	ND	15.80 (5 mg/mL)	[149]

Table S3 Acetylcholinesterase inhibitory effects of medicinal plants chosen from the list of Thai FDA-approved dietary supplements derived from medicinal plants.

No	Scientific name	Acetylcholinesterase (AChE)*		References
		IC_{50} (mg/mL)	%inhibition (tested conc.)	
22	*Capsicum annuum* (bird eye's chilli)	ND	ND	
23	*Carthamus tinctorius*	30.33	ND	[135]
24	*Centella asiatica*	ND	3.24 (1 mg/mL)	[140]
25	*Chaenomeles speciosa*	ND	ND	
26	*Chrysanthemum indicum*	0.03	ND	[150]
27	*Chrysanthemum morifolium*	ND	ND	
28	*Cinnamomum cassia*	ND	ND	
29	*Cinnamomum verum*	ND	0.5 (10 µL)	[142]
30	*Citrus aurantifolia*	0.02	ND	[151]
31	*Citrus aurantium*	0.15	ND	[152]
32	*Citrus limon*	0.85	ND	[153]
33	*Citrus reticulate*	ND	ND	
34	*Citrus sinensis*	ND	30.89 (0.2%v/v)	[154]
35	*Cocos nucifera*	ND	ND	
36	*Coffea arabica*	ND	0.95 (200 µL)	[155]
37	*Coix lacryma-jobi*	ND	0.38 (0.9 mg/mL)	[156]
38	*Cucumis melo*	ND	ND	
39	*Cucurbita pepo*	ND	ND	
40	*Curcuma longa*	0.06	96.5 (0.5 mM)	[157]
41	*Cymbopogon citratus*	ND	12.4 (0.2 mg/mL)	[137]
42	*Cynara scolymus*	0.09	ND	[158]
43	*Daucus carota*	13.13	ND	[159]

Table S3 Acetylcholinesterase inhibitory effects of medicinal plants chosen from the list of Thai FDA-approved dietary supplements derived from medicinal plants.

No	Scientific name	Acetylcholinesterase (AChE)*		References
		IC50 (mg/mL)	%inhibition (tested conc.)	
44	*Fragaria ananassa*	0.02	77.7 (0.5 mg/mL)	[160]
45	*Ganoderma lucidum*	ND	32.5 (1 mg/mL)	[161]
46	*Garcinia atroviridis*	0.03	80.2 (0.1 mg/mL)	[162]
47	*Garcinia mangostana*	ND	13.79 (1 mg/mL)	[140]
48	*Ginkgo biloba*	ND	8.08 (1 mg/mL)	[140]
49	*Glycine max*	ND	ND	
50	*Glycyrrhiza uralensis*	ND	ND	
51	*Gynostemma pentaphyllum*	ND	19.64 (2 mg/mL)	[147]
52	*Helianthus annuus*	ND	ND	
53	*Helianthus tuberosus*	ND	5.51 (1 mg/mL)	[140]
54	*Hibiscus sabdariffa*	ND	19.05 (2 mg/mL)	[147]
55	*Hippophae rhamnoides*	ND	75.9 (0.5 mg/mL)	[163]
56	*Hordeum vulgare*	ND	0.71 (35 µL)	[164]
57	*Houttuynia cordata*	0.08	ND	[165]
58	*Illicium verum*	0.06	ND	[166]
59	*Ipomoea aquatica*	0.17	62.54 (0.4 mg/mL)	[167, 168]
60	*Kaempferia parviflora*	ND	ND	
61	*Lentinula edodes*	ND	ND	
62	*Linum usitatissimum*	ND	ND	
63	*Lonicera japonica*	ND	ND	

Table S3 Acetylcholinesterase inhibitory effects of medicinal plants chosen from the list of Thai FDA-approved dietary supplements derived from medicinal plants.

No	Scientific name	Acetylcholinesterase (AChE)*		References
		IC_{50} (mg/mL)	%inhibition (tested conc.)	
64	*Lycium barbarum*	ND	0.71 (25 µL)	[169]
65	*Lycopersicon esculentum*	5.70	ND	[170]
66	*Malus domestica*	ND	5.87 (1 mg/mL)	[140]
67	*Matricaria chamomilla*	0.60	ND	[171]
68	*Medicago sativa*	0.02	ND	[172]
69	*Melissa officinalis*	ND	1.72 (25 µL)	[173]
70	*Mentha* x *piperita*	ND	4.2 (10 µL)	[142]
71	*Mentha spicata*	0.02	ND	[174]
72	*Momordica cochinchinensis*	ND	9.17 (1 mg/mL)	[140]
73	*Momordica grosvenorii*	ND	ND	
74	*Moringa oleifera*	0.21	ND	[175]
75	*Morus alba*	ND	4.24 (1 mg/mL)	[140]
76	*Musa sapientum*	29.14	ND	[135]
77	*Myrciaria dubia*	1.57	ND	[176]
78	*Ocimum basilicum*	0.65	ND	[177]
79	*Opuntia ficus-indica*	0.78	ND	[178]
80	*Origanum vulgare*	0.003	95.61 (0.25 mg/mL)	[179]
81	*Oryza sativa*	ND	13.27 (1 mg/mL)	[140]
82	*Paeonia lactiflora*	ND	ND	
83	*Panicum miliaceum*	ND	ND	
84	*Persea americana*	ND	ND	

Table S3 Acetylcholinesterase inhibitory effects of medicinal plants chosen from the list of Thai FDA-approved dietary supplements derived from medicinal plants.

No	Scientific name	Acetylcholinesterase (AChE)*		References
		IC_{50} (mg/mL)	%inhibition (tested conc.)	
85	*Petroselinum crispum*	ND	21.00 (0.1 mg/mL)	[180]
86	*Phaseolus mungo*	ND	ND	
87	*Phaseolus vulgaris*	ND	ND	
88	*Phyllanthus emblica*	ND	17.36 (1 mg/mL)	[140]
89	*Pinus massoniana*	ND	ND	
90	*Piper nigrum*	58.02	ND	[135]
91	*Piper sarmentosum*	ND	73.61 (0.1 mg/mL)	[181]
92	*Pisum sativum*	ND	32.00 (0.1 mg/mL)	[182]
93	*Pleurotus ostreatus*	1.75	ND	[139]
94	*Plukenetia volubilis*	ND	ND	
95	*Prunus armeniaca*	0.45	ND	[183]
96	*Prunus cerasus*	ND	ND	
97	*Prunus domestica*	18.07	ND	[184]
98	*Prunus mume*	ND	ND	
99	*Punica granatum*	0.08	62.4 (1 mg/mL)	[143]
100	*Rosa centifolia*	ND	ND	
101	*Rosmarinus officinalis*	ND	17.00 (0.1 mg/mL)	[180]
102	*Rubus fruticosus*	331.02	47.82 (320 mg/mL)	[185]
103	*Rubus idaeus*	ND	ND	
104	*Saccharum officinarum*	ND	ND	
105	*Salvia hispanica*	0.02	38.82 (10 μg/mL)	[186]

Table S3 Acetylcholinesterase inhibitory effects of medicinal plants chosen from the list of Thai FDA-approved dietary supplements derived from medicinal plants.

No	Scientific name	Acetylcholinesterase (AChE)*		References
		IC_{50} (mg/mL)	%inhibition (tested conc.)	
106	*Sesamum indicum*	ND	66.17	[187]
107	*Solanum torvum*	ND	1.8 (50 µL)	[188]
108	*Tagetes erecta*	1.13	ND	[189]
109	*Terminalia chebula*	0.19	41.06 (1 mg/mL)	[143]
110	*Thymus vulgaris*	0.22	ND	[153]
111	*Trigonella-foenum graecum*	ND	6.00 (1 mg/mL)	[143]
112	*Triticum aestivum*	ND	ND	
113	*Vaccinium macrocarpon*	ND	ND	
114	*Vitis vinifera*	ND	3.86 (1 mg/mL)	[190]
115	*Vigna radiata*	ND	ND	
116	*Zingiber officinale*	ND	0.6 (10 µL)	[142]

*ND; No data available

References

1. Bekhradnia S, Nabavi SM, Nabavi SF, Ebrahimzadeh MA. Antioxidant activity of kiwifruit (*Actinidia chinensis*). Pharmacology Online. 2011; 1: 758–764.
2. Salama AZ, Aboul-Enein MA, Gaafar AA, Abou-Elella F, Aly FH, Asker SM, *et al*. Active Constituents of Kiwi (*Actinidia Deliciosa* Planch) Peels and Their Biological Activities as Antioxidant, Antimicrobial and Anticancer. Res J Chem Environ. 2018 Sep; 9(22):52-59.
3. Rajan S, Gokila M, Jency P, Brindha P, Sujatha RK. Antioxidant and phytochemical properties of *Aegle marmelos* fruit pulp. Int J Curr Pham Res. 2011;3(2):65-70.
4. Dash PK, Das S, Mannan MA, Jahan M. Comparative evaluation of onion germplasm (*Allium cepa* L.) of Bangladesh for physical and antioxidant properties. SAARC J Agric. 2021;19(1):113-123. Doi: https://doi.org/10.3329/sja.v19i1.54783
5. Ye CL, Dai DH, Hu WL. Antimicrobial and antioxidant activities of the essential oil from onion (*Allium cepa* L.). Food control. 2013;30(1):48-53. Doi: https://doi.org/10.1016/j.foodcont.2012.07.033
6. Chang TC, Chang HT, Chang ST, Lin SF, Chang YH, Jang HD. A comparative study on the total antioxidant and antimicrobial potentials of ethanolic extracts from various organ tissues of *Allium* spp. Food Nutr Sci. 2013;4(8):182-190. Doi: https://doi.org/10.4236/fns.2013.48a022
7. Zhao Q, Xie B, Yan J, Zhao F, Xiao J, Yao L, et al. *In vitro* antioxidant and antitumor activities of polysaccharides extracted from *Asparagus officinalis*. Carbohydr Polym. 2012;87(1):392-396. Doi: https://doi.org/10.1016/j.carbpol.2011.07.068
8. Kim GH, Duan Y, Lee SC, Kim HS. Assessment of antioxidant activity of garlic (*Allium sativum* L.) peels by various extraction solvents. J of Korean Oil Chemists' Soc. 2016;33(1):204-212. Doi: https://doi.org/10.12925/jkocs.2016.33.1.204
9. Mazzulla S, Sesti S, Schella A, Perrotta I, Anile A, Drogo S. Protective effect of *Aloe vera (Aloe barbadensis* Miller*)* on erythrocytes anion transporter and oxidative change. Food Nutr Sci. 2012;3(12):1697-1702 Doi: https://doi.org/10.4236/fns.2012.312222
10. Uddin MN, Roy SC, Mamun AA, Mitra K, Haque MZ, Hossain ML. Phytochemicals and *in-vitro* antioxidant activities of *Aloe vera* gel. J Bangladesh Acad Sci. 2020;44(1):33-41. Doi: https://doi.org/10.3329/jbas.v44i1.48561
11. Mahae N, Chaiseri S. Antioxidant Activities and Antioxidative Components in Extracts of *Alpinia galanga* (L.) Sw. Kasetsat J (Nat Sci).2009;43(2):358-369.
12. Srividya AR, Dhanabal SP, Satish kumar MN, Parth kumar HB. Antioxidant and antidiabetic activity of *Alpinia galangal*. Int J Pharm Res. 2010;3(1):6-12.
13. Fidrianny I, Virna V, Insanu M. Antioxidant potential of different parts of Bogor Pineapple (*Ananas comosus* (L.) merr. var. queen) cultivated in West Java-Indonesia. Asian J Pharm Clin Res. 2018;11(1):129-133. Doi: https://doi.org/10.22159/ajpcr.2018.v11i1.22022
14. Putri DA, Ulfi A, Purnomo AS, Fatmawati S. Antioxidant and antibacterial activities of *Ananas comosus* peel extracts. Mal J Fund Appl Sci. 2018;14(2):307-311.
15. Makchuchit S, Itharat A, Tewtrakul S. Antioxidant and nitric oxide inhibition activities of Thai medicinal plants. J Med Assoc Thai. 2010 Dec;93:S237-35. PMID: 21294419.
16. Li SY, Yu Y, Li SP. Identification of antioxidants in essential oil of radix *Angelicae sinensis* using HPLC coupled with DAD-MS and ABTS-based assay. J Agric Food Chem. 2007;55(9):3358-3362. Doi: https://doi.org/10.1021/jf070140t
17. Gargantiel MF, Ysrael MC. Antioxidant activity and hypoglycemic potential of *Antidesma ghaesembilla* Gaertn (Phyllantaceae). Int J Sci Technol Res. 2014;3(3):422-431.
18. Ud din Z, Shad AA, Bakht J, Ullah I, Jan S. *In vitro* antimicrobial, antioxidant activity and phytochemical screening of *Apium graveolens*. Pak J Pharm Sci. 2015;28(5):1600-1704.

19. Moreno-Escobar JA, Bazaldúa S, Villarreal ML, Bonilla-Barbosa JR, Mendoza S, Rodríguez-López V. Cytotoxic and antioxidant activities of selected Lamiales species from Mexico. Pharm Biol. 2011;49(12):1243-1258. Doi: https://doi.org/10.3109/13880209.2011.589454
20. Alonzo-Macías M, Cardador-Martínez A, Besombes C, Allaf K, Tejada-Ortigoza V, Soria-Mejía MC, *et al*. Instant controlled pressure drop as blanching and texturing pre-treatment to preserve the antioxidant compounds of red dried beetroot (*Beta vulgaris* L.). Molecules. 2020;25(18):4132. Doi: https://doi.org/10.3390/molecules25184132
21. Mat Yasin ZA, Khazali AS, Ibrahim F, Nor Rashid N, Yusof R. Antioxidant and enzyme inhibitory activities of *Areca catechu*, *Boesenbergia Rotunda*, *Piper Betle* and *Orthosiphon aristatus* for potential skin anti-aging properties. Curr Top Nutraceutical Res. 2018;17(3):229-235. Doi: https://doi.org/10.37290/ctnr2641-452x.17:229-235
22. Leamsamrong K, Tongjaroenbuangam W, Maneetong S, Chantiratikul A, Chinrasri O, Chantiratikul P. Physicochemical contents, antioxidant activities, and acute toxicity assessment of selenium-enriched Chinese kale (*Brassica oleracea* var. *alboglabra* L.) seedlings. J Chem. 2019;2019:1-12. Doi: https://doi.org/10.1155/2019/7983038
23. Khongrum J, Yingthongchai P, Tateing S. Exploring the synergistic effect of celery and Chinese kale on antioxidant and anti-pancreatic lipase and anti-acetylcholinesterase activities. Open Acc J Bio Sci. 2021;3(2):899-904. Doi: https://doi.org/10.38125/oajbs.000264
24. Yang SR, Songzhuzhao, Boo HO. Antioxidant activity of several cabbage (*Brassica oleracea* L.) cultivars. Korean J Plant Res. 2015;28(3):312-320. Doi: https://doi.org/10.7732/kjpr.2015.28.3.312
25. Chaudhary A, Choudhary S, Sharma U, Vig AP, Singh B, Arora S. Purple head broccoli (*Brassica oleracea* L. var. *italica* Plenck), a functional food crop for antioxidant and anticancer potential. J Food Sci Technol. 2018;55(5):1806-1815. Doi: https://doi.org/10.1007/s13197-018-3095-0
26. Sri Agustini K, Sri Agustini NW. Potential lutein extract of broccoli (*Brassica oleracea* L. var. Italica) as antiradical ABTS (2,2 - azinobis acid, 3-ethyl benzothiazoline-6-sulfonic acid). In ICBS Conference Proceedings Conference on Biological Science (2015), KnE Life Sciences; 2015. P. 125–135.
27. Preethi KC, Kuttan G, Kuttan R. Antioxidant Potential of an Extract of *Calendula officinalis* flowers *in vitro* and *in vivo*. Pharm Biol. 2006;44(9):691-697. Doi: https://doi.org/10.1080/13880200601009149
28. Pereira VP, Knor FJ, Vellosa JCR, Beltrame FL. Determination of phenolic compounds and antioxidant activity of green, black and white teas of *Camellia sinensis* (L.) kuntze. Rev Bras plants med. 2014;16(3):490-498. Doi: https://doi.org/10.1590/1983-084x/13_061
29. Loizzo MR, Pugliese A, Bonesi M, Menichini F, Tundis R. Evaluation of chemical profile and antioxidant activity of twenty cultivars from *Capsicum annuum*, *Capsicum baccatum*, *Capsicum chacoense* and *Capsicum chinense*: A comparison between fresh and processed peppers. LWT - Food Sci Technol. 2015;64(2):623-631. Doi: https://doi.org/10.1016/j.lwt.2015.06.042
30. Olatunji TL, Afolayan AJ. Comparative quantitative study on phytochemical contents and antioxidant activities of *Capsicum annuum* L. and *Capsicum frutescens* L. The Sci World J. 2019;2019:1-13. Doi: https://doi.org/10.1155/2019/4705140
31. Sun LP, Shi FF, Zhang WW, Zhang ZH, Wang K. Antioxidant and anti-inflammatory activities of safflower (*Carthamus Tinctorius* L.) honey extract. Food. 2020;9(8):1039. Doi: https://doi.org/10.3390/foods9081039
32. Mohapatra P, Ray A, Jena S, Nayak S, Mohanty S. Influence of extraction methods and solvent system on the chemical composition and antioxidant activity of *Centella asiatica* L. leaves. Biocatal Agric Biotechnol. 2021;33:101971. Doi: https://doi.org/10.1016/j.bcab.2021.101971
33. Tian BM, Xie XM, Shen PP, Wu J, Wang J. Comparison of the antioxidant activities and the chemical compositions of the antioxidants of different polarity crude extracts from the fruits of *Chaenomeles speciose* (sweet) Nakai. J Phys Chem Lett. 2015;28(6):443-447. https://doi.org/10.1556/1006.2015.28.6.4
34. Turkiewicz IP, Wojdyło A, Tkacz K, Nowicka P, Golis T, Bąbelewski P. ABTS on-line antioxidant, α-amylase, α-glucosidase, pancreatic lipase, acetyl- and butyrylcholinesterase inhibition activity of Chaenomeles fruits

determined by polyphenols and other chemical compounds. Antioxidants. 2020;9(1):60. Doi: https://doi.org/10.3390/antiox9010060
35. Youssef FS, Eid SY, Alshammari E, Ashour ML, Wink M, El-Readi MZ. *Chrysanthemum indicum* and *Chrysanthemum morifolium*: Chemical composition of their essential oils and their potential use as natural preservatives with antimicrobial and antioxidant activities. Food. 2020;9(10):1460. Doi: https://doi.org/10.3390/foods9101460
36. Yuan Q, Fu Y, Xiang PY, Zhao L, Wang SP, Zhang Q, *et al*. Structural characterization, antioxidant activity, and antiglycation activity of polysaccharides from different *Chrysanthemum* teas. RSC Adv. 2019;9(61):35443-35451. Doi: https://doi.org/10.1039/c9ra05820f
37. Gong J, Chu B, Gong L, Fang Z, Zhang X, Qiu S, *et al*. Comparison of phenolic compounds and the antioxidant activities of fifteen *Chrysanthemum morifolium* Ramat CV. 'Hangbaiju' in China. Antioxidants. 2019;8(8):325. Doi: https://doi.org/10.3390/antiox8080325
38. Brodowska KM, Brodowska AJ, Śmigielski K, Łodyga-Chruścińska E. Antioxidant profile of essential oils and extracts of cinnamon bark (*Cinnamomum cassia*). Eur J Biol. 2016;6(4):310-316.
39. Gulcin I, Kaya R, Goren AC, Akincioglu H, Topal M, Bingol Z, *et al*. Anticholinergic, antidiabetic and antioxidant activities of cinnamon (*Cinnamomum verum*) bark extracts. Int J Food Prop. 2019;22(1):1511-1526. Doi: https://doi.org/10.1080/10942912.2019.1656232
40. Lin LY, Chuang CH, Chen HC, Yang KM. Lime (*Citrus aurantifolia* (Christm.) Swingle) essential oils: Volatile compounds, antioxidant capacity, and hypolipidemic effect. Food. 2019;8(9):398. Doi: https://doi.org/10.3390/foods8090398
41. Zeghad N, Ahmed E, Belkhiri A, Heyden YV, Demeyer K. Antioxidant activity of *Vitis vinifera, Punica granatum, Citrus aurantium* and *Opuntia Ficus* indica fruits cultivated in Algeria. Heliyon. 2019;5(4):e01575. Doi: https://doi.org/10.1016/j.heliyon.2019.e01575
42. Loizzo M, Sicari V, Tundis R, Leporini M, Falco T, Calabrò V. The influence of ultrafiltration of *Citrus limon* L. Burm. CV *Femminello comune* juice on its chemical composition and antioxidant and hypoglycemic properties. Antioxidants. 2019;8(1):23. Doi: https://doi.org/10.3390/antiox8010023
43. Singanusong R, Nipornram S, Tochampa W, Rattanatraiwong P. Low power ultrasound-assisted extraction of phenolic compounds from Mandarin (*Citrus reticulata* Blanco CV. Sainampueng) and lime (*Citrus aurantifolia*) peels and the Antioxidant. Food Anal. Methods. 2015;8(5):1112-1123. Doi: https://doi.org/10.1007/s12161-014-9992-6
44. Lin X, Cao S, Sun J, Lu D, Zhong B, Chun J. The chemical compositions, and antibacterial and antioxidant activities of four types of citrus essential oils. Molecules. 2021;26(11):3412. Doi: https://doi.org/10.3390/molecules26113412
45. Siramon P, Wongsheree T, Yuadyong S. Ultrasound-assisted extraction of phenolic compounds from coconut endocarp and its radical scavenging activity. Naresuan Phayao J. 2020;13(3):22-28.
46. Kaisangsri N, Selamassakul O, Sonklin C, Laohakunjit N, Kerdchoechuen O, Rungruang, R. Phenolic compounds and biological activities of coffee extract for cosmetic product. SEATUC J Sci Eng. 2019;1(1):71-76.
47. Yuan J, Liang Y, Cui S, Zhang X, Wang L, Qiao Y. Angiotensin Ⅰ converting enzyme inhibitory and antioxidant activity of adlay (*Coix lacryma-jobi* L. Var. Ma-yuen stapf) glutelin hydrolysate. Ital J Food Sci. 2014;26(3):282-288.
48. Wu Z, Zhang M, Yang H, Zhou H, Yang H. Production, physico-chemical characterization and antioxidant activity of natural melanin from submerged cultures of the mushroom *Auricularia auricula*. Food Biosci. 2018;26:49-56. Doi: https://doi.org/10.1016/j.fbio.2018.09.008
49. Widowati W, Widyanto RM, Laksmitawati DR, Erawijantari PP, Wijaya L, Sandra F. Phytochemical, free radical scavenging and cytotoxic assay of *Cucumis melo* L. extract and β-carotene. J Adv Agric Technol. 2015;2(2):114-119. Doi: https://doi.org/10.12720/joaat.2.2.114-119
50. Hayet E, Liouane K, Thabti F, Skhir F, Aouni M, Mastour M. *In vitro* anticandidal, antiviral and antioxidant activities of *Cucumis melo* L. var. *Cantalupensis naud* extracts. J Food Nutr Res. 2016;4(9):596-599.

51. Thangavel S, Kasiramar G, Sivakumar A. Antioxidant potential and Phytochemical analysis of fruit extract of *Cucurbita pepo*. Int. J. Curr. Res. Chem. Pharm. Sci. 2019;6(3):22-32. Doi: https://doi.org/10.22192/ijcrcps.2019.06.03.003
52. Tanvir EM, Hossen MS, Hossain MF, Afroz R, Gan SH, Khalil MI. Antioxidant properties of popular turmeric *(Curcuma longa)* varieties from Bangladesh. J Food Qual. 2017. Doi: https://doi.org/10.1155/2017/8471785
53. Mošovská S, Petáková P, Kaliňák M, Mikulajová A. Antioxidant properties of curcuminoids isolated from *Curcuma longa* L. Acta Chim Slov. 2016;9(2):130-135. Doi: https://doi.org/10.1515/acs-2016-0022
54. Salaria D, Rolta R, Sharma N, Dev K, Sourirajan A, Kumar V. *In silico* and *in vitro* evaluation of the anti-inflammatory and antioxidant potential of *Cymbopogon citratus* from north-western Himalayas. *BioRxiv*. 2020. Doi: https://doi.org/10.1101/2020.05.31.124982
55. Kollia E, Markaki P, Zoumpoulakis P, Proestos C. Comparison of different extraction methods for the determination of the antioxidant and antifungal activity of *Cynara scolymus* and *C. cardunculus* extracts and infusions. Nat Prod Commun. 2017;12(3):423-426. Doi: https://doi.org/10.1177/1934578x1701200329
56. Bueno-Gavilá E, Abellán A, Girón-Rodríguez F, Cayuela JM, Tejada L. Bioactivity of hydrolysates obtained from chicken egg ovalbumin using artichoke (*Cynara Scolymus* L.) proteases. Foods. 2021;10(2):246. Doi: https://doi.org/10.3390/foods10020246
57. Servi H, Şen A, Yildirim servi E, Doğan A. Chemical composition and biological activities of essential oils of *Foeniculum vulgare* mill. and *Daucus carota* L. growing wild in Turkey. J Res Pharm. 2021;25(2):142-152. Doi: https://doi.org/10.29228/jrp.5
58. Soulef S, Seddik K, Nozha M, Smain A, Saliha D, Karim H. Phytochemical screening and *in vivo* and *in vitro* evaluation antioxidant capacity of *Fargaria ananassa*, *Prunus armeniaca* and *Prunus persica* fruits growing in Algeria. Prog Nutr. 2020;22(1):236-252. Doi: https://doi.org/10.23751/pn.v22i1.8011
59. Tel G. Ozturk M, Duru ME, Turkoglu A. Antioxidant and anticholinesterase activities of five wild mushroom species with total bioactive contents. Pharm Biol. 2015;53(6):824-830. Doi: https://doi.org/10.3109/13880209.2014.943245
60. Chatatikun M, Supjaroen P, Promlat P, Chantarangkul C, Waranuntakul S, Nawarat J, *et al*. Antioxidant and tyrosinase inhibitory properties of an aqueous extract of *Garcinia atroviridis* Griff. ex. T. Anderson Fruit Pericarps. Pharmacogn J. 2020;12(1):71-78. Doi: https://doi.org/10.5530/pj.2020.12.12
61. Mohammad NA, Abang Zaidel DN, Muhamad II, Abdul Hamid M, Yaakob H, Mohd Jusoh YM. Optimization of the antioxidant-rich xanthone extract from Mangosteen (*Garcinia Mangostana* L.) pericarp via microwave-assisted extraction. Heliyon. 2019;5(10). Doi: https://doi.org/10.1016/j.heliyon.2019.e02571
62. Qa'dan F, Mansoor K, AL-Adham I, Schmidt M, Nahrstedt A. Proanthocyanidins from *Ginkgo biloba* leaf extract and their radical scavenging activity. Pharm Biol. 2011;49(5):471-476. Doi: https://doi.org/10.3109/13880209.2010.523831
63. Li L, Zhang MX, Wang XY, Yang YL, Gong X, Wang CC, *et al*, Assessment of components of *Gingko biloba* leaves collected from different regions of China that contribute to its antioxidant effects for improved quality monitoring. Food Sci Technol. 2021;41(2):676-683.
64. Prahastuti S, Hidayat M, Hasianna ST, Widowati W, Amalia A, Yusepany DT, *et al*. Antioxidant potential ethanolic extract of *Glycine max* (L.) Merr. Var. Detam and daidzein. J Phy Conf Ser. 2019;1374. Doi: https://doi.org/10.1088/1742-6596/1374/1/012020
65. Fan R, Li N, Jiang X, Yuan F, Gao Y. HPLC–Dad–MS/Ms Identification and HPLC–ABTS$^{·+}$ on-line antioxidant activity evaluation of bioactive compounds in liquorice (*Glycyrrhiza uralensis* Fisch.) extract. Eur Food Res Technol. 2014;240(5):1035-1048. Doi: https://doi.org/10.1007/s00217-014-2407-5
66. Wang TX, Shi MM, Jiang JG. Bioassay-guided isolation and identification of anticancer and antioxidant compounds from *Gynostemma pentaphyllum* (thunb.) Makino. RSC Adv. 2018;8(41):23181-23190. Doi: https://doi.org/10.1039/c8ra02803f

67. Islam RT, Islam AT, Hossain MM, Mazumder K. *In vivo* analgesic activity of methanolic extract of *Helianthus annuus* seeds. Int Curr Pharm J. 2016;5(4):38-40. Doi: https://doi.org/10.3329/icpj.v5i4.27019
68. Kusmiati, Ningsih EB, Ramadhani I, Amir M. Antibacterial and antioxidant activity test of crude lutein extracted from sunflower (*Helianthus annuus* L.). In AIP Conference Proceedings 2021 Apr 2 (Vol. 2331, No. 1). AIP Publishing. Doi: https://doi.org/10.1063/5.0041594
69. Mariadoss AV, Park SJ, Saravanakumar K, Sathiyaseelan A, Wang MH. Ethyl acetate fraction of *Helianthus tuberosus* L. induces anti-diabetic, and wound-healing activities in insulin-resistant human liver cancer and mouse fibroblast cells. Antioxidants. 2021;10(1):99. Doi: https://doi.org/10.3390/antiox10010099
70. Yang L, Gou Y, Zhao T, Li F, Zhang B, Wu X. Antioxidant capacity of extracts from calyx fruits of roselle (*Hibiscus sabdariffa* L.). Afr J Biotechnol. 2012;11(17):4063-4068. Doi: https://doi.org/10.5897/AJB11.2227
71. Kant V, Mehta M, Varshneya C. Antioxidant potential and total phenolic contents of Sea buckthorn (*Hippophae rhamnoides*) pomace. Free Rad Antiox. 2012;2(4):79-86. Doi: https://doi.org/10.5530/ax.2012.4.14
72. Boubakri H, Jdey A, Taamalli A, Taamalli W, Jebara M, Brini F, *et al*. Phenolic composition as measured by liquid chromatography/ mass spectrometry and biological properties of Tunisian barley. Int J Food Prop. 2017;20(2):1783-1797. Doi: https://doi.org/10.1080/10942912.2017.1359186
73. Tuyen P, Anh TTT, Pham T, Tan KD. Antioxidant properties and total phenolic contents of various extracts from *Houttuynia cordata* Thunb. Acad J Biol. 2018;40(2se):149-154. Doi: https://doi.org/10.15625/0866-7160/v40n2se.11683
74. Rao KS, Keshar NK, Kumar BVVR. A comparative study of polyphenolic composition and *in vitro* antioxidant activity of *Illicium verum* extracted by microwave and soxhlet extraction techniques. Ind J Pharm Edu Res. 2012;46(3):228-234.
75. Malakar C, Choudhury PPN. Pharmacological potentiality and medicinal uses of *Ipomoea aquatica* Forsk: a review. Asian J Pharm Clin Res. 2015;8(2):60-63.
76. Choi MH, Kim KH, Yook HS. Antioxidant activity of fermented *Kaempferia parviflora* and inhibitory action against tyrosinase and elastase. J Korean Soc Food Nutr. 2018;47(11):1076-1084. Doi: https://doi.org/10.3746/jkfn.2018.47.11.1076
77. Elhusseiny SM, El-Mahdy TS, Awad MF, Elleboudy NS, Farag MM, Yassein MA, *et al*. Proteome analysis and *in vitro* antiviral, anticancer and antioxidant capacities of the aqueous extracts of *Lentinula edodes* and *Pleurotus ostreatus* edible mushrooms. Molecules. 2021;26(15):4623. Doi: https://doi.org/10.3390/molecules26154623
78. Han H, Yılmaz H, Gülçin İ. Antioxidant activity of Flaxseed (*Linum usitatissimum* L.) shell and analysis of its polyphenol contents by LC-MS/MS. Rec Nat Prod. 2018;12(4):397-402. Doi: https://doi.org/10.25135/rnp.46.17.09.155
79. Fan ZL, Li L, Bai XL, Zhang H, Liu QR, Zhang *et al*. Extraction optimization, antioxidant activity, and tyrosinase inhibitory capacity of polyphenols from *Lonicera japonica.* Food Sci Nutr. 2019;7(5):1786-1794. Doi: https://doi.org/10.1002/fsn3.1021
80. Skenderidis P, Kerasioti E, Karkanta E, Stagos D, Kouretas D, Petrotos K, *et al*. Assessment of the antioxidant and antimutagenic activity of extracts from Goji Berry of greek cultivation. Toxicol Rep. 2018;5:251-257. Doi: https://doi.org/10.1016/j.toxrep.2018.02.001
81. Kim S, Choi WH, Ahn J, Ha TY. Antioxidative Activity of Cherry Tomato (*Lycopersicon lycopersicum* var. *cerasiforme*) Extracts and Protective Effect for H_2O_2 -induced Inhibition of Gap Junction Intercellular Communication. Food Sci Biotechnol. 2009;18(3):630-635.
82. Fidrianny I, Fitrani D, Kusmardiyani S, Rizaldy D, Ruslan K. *In vitro* antioxidant and phytochemical analysis of different parts of SIMANA lagi apple (*Malus domestica* Borkh. “Simana Lagi”) grown in East Java-Indonesia. Asian J Pharm Clin Res. 2017;10(11:41. Doi: https://doi.org/10.22159/ajpcr.2017.v10i11.20649

83. Hajaji S, Alimi D, Jabri MA, Abuseir S, Gharbi M, Akkari H. Anthelmintic activity of Tunisian chamomile (*Matricaria recutita* L.) against *Haemonchus contortus*. J Helminthol. 2017;92(2):168-177. Doi: https://doi.org/10.1017/s0022149x17000396
84. Rana MG, Katbamna RV, Padhya AA, Dudhrejiya AD, Jivani NP, Sheth NR. *In vitro* antioxidant and free radical scavenging studies of alcoholic extract of *Medicago sativa* L. Rom J Biol Plant Biol. 2010;55(1):15-22.
85. Rădulescu M, Jianu C, Lukinich-Gruia AT, Mioc M, Mioc A, Șoica C, *et al*. Chemical composition, *in vitro* and *in silico* antioxidant potential of *Melissa officinalis* subsp. officinalis essential oil. Antioxidants. 2021;10(7):1081. Doi: https://doi.org/10.3390/antiox10071081
86. Nickavar B, Alinaghi A, Kamalinejad M. Evaluation of the Antioxidant Properties of Five Mentha Species. Int J Pharm Res. 2008;7(3):203-209.
87. Ismail A, Abdulqader A, Ali F, Esa NM Antioxidant compounds and capacities of GAC (*Momordica cochinchinensis* spreng) fruits. Asian Pac J Trop Biomed. 2019;9(4):158. Doi: https://doi.org/10.4103/2221-1691.256729
88. Wimalasiri DC. Genetic diversity, nutritional and biological activity of *Momordica cochinensis* (Cucurbitaceae) (Doctor of Philosophy's). RMIT University.
89. Liu H, Wang C, Qi X, Zou J, Sun Z. Antiglycation and antioxidant activities of mogroside extract from *Siraitia grosvenorii* (Swingle) fruits. J Food Sci Technol. 2018;55(5):1880-1888. Doi: https://doi.org/10.1007/s13197-018-3105-2
90. Xu YB, Chen GL, Guo MQ. Antioxidant and anti-inflammatory activities of the crude extracts of *Moringa oleifera* from Kenya and their correlations with flavonoids. Antioxidants. 2019;8(8):296. Doi: https://doi.org/10.3390/antiox8080296
91. Thabti I, Elfalleh W, Tlili N, Ziadi M, Campos MG, Ferchichi A. Phenols, flavonoids, and antioxidant and antibacterial activity of leaves and stem bark of *Morus* Species. Int J Food Prop. 2013; 17(4):842-854. Doi: https://doi.org/10.1080/10942912.2012.660722
92. Dahham SS, Agha MT, Tabana YM, Abdul Majid AMS. Antioxidant activities and anticancer screening of extracts from banana fruit (*Musa sapientum*). Academic J Cancer Res. 2015;8:28-34.
93. Fidrianny I, KikiRizki R, Insanu M. *In vitro* antioxidant activities from various extracts of banana peels using ABTS, DPPH assays and correlation with phenolic, flavonoid, and carotenoid content. Int J Pharm Pharm Sci. 2014;6(8):299-303.
94. Castro JC, Maddox JD, Cobos M, Imán SA. *Myrciaria dubia* "camu camu" fruit: Health-promoting phytochemicals and functional genomic characteristics. Breeding and Health Benefits of Fruit and Nut Crops. 2018. Doi: https://doi.org/10.5772/intechopen.73213
95. Sriram GP, Santhana PB, Subramanian S. Antioxidant Properties of *Ocimum basilicum* Leaves Extract: An *in vitro* study. Der Pharmacia Lettre. 2019;11(1):33-41.
96. Zeghad N, Ahmed E, Belkhiri A, Heyden YV, Demeyer K. Antioxidant activity of *Vitis vinifera*, *Punica granatum*, *Citrus aurantium* and *Opuntia Ficus* indica fruits cultivated in Algeria. Heliyon. 2019;5(4). Doi: https://doi.org/10.1016/j.heliyon.2019.e01575
97. Kaska A. The phenolic content, antioxidant and cytotoxic activities of *Origanum sipyleum* from Turkey. Int J Sec Metabolite. 2018;5(4):343-352. Doi: https://doi.org/10.21448/ijsm.468418
98. Surin S, You SG, Seesuriyachan P, Muangrat R, Wangtueai S, Jambrak AR, *et al*. Optimization of ultrasonic-assisted extraction of polysaccharides from purple glutinous rice bran (*Oryza sativa* L.) and their antioxidant activities. Sci Rep. 2020;10(1):10410. Doi: https://doi.org/10.1038/s41598-020-67266-1
99. Meng J, Cheng M, Liu L, Sun J, Condori-Apfata JA, Zhao D. *In-vitro* antioxidant and *in-vivo* anti-aging with stress resistance on *Caenorhabditis elegans* of *Herbaceous peony* stamen tea. Int J Food Prop. 2021;24(1):1349-1366. Doi: https://doi.org/10.1080/10942912.2021.1967385
100. Ghimire B, Yu C, Kim S, Chung IM. Diversity in accessions of *Panicum miliaceum* L. based on agro-morphological, Antioxidative, and genetic traits. Molecules. 2019;24(6):1012. Doi: https://doi.org/10.3390/molecules24061012

101. Antasionasti I, Riyanto S, Rohman A. Antioxidant activities and phenolics contents of avocado (*Persea americana* mill.) peel *in vitro*. Res J Med Plants. 2017;11(2):55-61. Doi: https://doi.org/10.3923/rjmp.2017.55.61
102. Yu M, Gouvinhas I, Rocha J, Barros AI. Phytochemical and antioxidant analysis of medicinal and food plants towards bioactive food and pharmaceutical resources. Sci Rep. 2021;11(1). Doi: https://doi.org/10.1038/s41598-021-89437-4
103. Saeed SM, Ali SA, Ali R, Naz S, Sayeed SA, Mobin L, *et al*. Utilization of *Vigna mungo* flour as fat mimetic in biscuits: Its impact on antioxidant profile, polyphenolic content, storage stability, and quality attributes. Legum Sci. 2020;2(4). Doi: https://doi.org/10.1002/leg3.58
104. Khatun S, Kim T. Phenolic compound, antioxidant activity and nutritional components of five legume seed. Am J Biomed Sci Res. 2021;12(4):328-334.
105. Abdulrahman BO, Bala M, Oluwasesan MB. Evaluation of *in vitro* antioxidant and antidiabetic potential of extracts from *Phaseolus vulgaris* L. seeds (black turtle beans). Funct Food Sci. 2021;1(9):23. Doi: https://doi.org/10.31989/ffs.v1i9.821
106. Charoenteeraboon J, Wongnoppavich A, Soonthornchareonnon N, Jaijoy K. Antioxidant activities of the standardized water extract from fruit of *Phyllanthus emblica* Linn. Songklanakarin J Sci Technol. 2010;32(6):599-604.
107. Ferreira-Santos P, Genisheva Z, Botelho C, Santos J, Ramos C, Teixeira JA, *et al*. Unraveling the biological potential of *Pinus pinaster* bark extracts. Antioxidants. 2020;9(4):334. Doi: https://doi.org/10.3390/antiox9040334
108. Akbar PN, Jahan IA, Hossain MH, Banik R, Nur HP, Hossain MT. Antioxidant capacity of *Piper longum* and *Piper nigrum* fruits grown in Bangladesh. World J Pharm Sci. 2014;2(9):890-1133.
109. Nguyen NQ, Nguyen VT, Van NT, Vo TN. Bioactive compounds and antioxidant activity of leaves from *Piper sarmentosum* Piperaceae. In IOP Conference Series: Materials Science and Engineering. 2020;99(1):p.012028. Doi: https://doi.org/10.1088/1757-899x/991/1/012028
110. Hadrich F, Arbi ME, Boukhris M, Sayadi S, Cherif S. Valorization of the peel of PEA: *Pisum sativum* by evaluation of its antioxidant and antimicrobial activities. J Oleo Sci. 2014;63(11):1177-1183. Doi: https://doi.org/10.5650/jos.ess14107
111. Elhusseiny SM, El-Mahdy TS, Awad MF, Elleboudy NS, Farag MM, Yassein MA, *et al*. Proteome analysis and *in vitro* antiviral, anticancer and antioxidant capacities of the aqueous extracts of *Lentinula edodes* and *Pleurotus ostreatus* edible mushrooms. Molecules. 2021;26(15):4623. Doi: https://doi.org/10.3390/molecules26154623
112. Wuttisin N, Nararatwanchai T, Sarikaputi A. Total phenolic, flavonoid, flavonol contents and antioxidant activity of Inca peanut (*Plukenetia volubilis* L.) leaves extracts. Food Res. 2020;5(1)216-224. Doi: https://doi.org/10.26656/fr.2017.5(1).346
113. Bonesi M, Tenuta M, Loizzo M, Sicari V, Tundis R. Potential application of *Prunus armeniaca* L. and *P. domestica* L. Leaf essential oils as antioxidant and of cholinesterases inhibitors. Antioxidants. 2018;8(1):2. Doi: https://doi.org/10.3390/antiox8010002
114. Becker M, Nunes G, Ribeiro D, Silva F, Catanante G, Marty JL. Determination of the antioxidant capacity of red fruits by miniaturized spectrophotometry assays. J Braz Chem Soc. 2019;30(5):1108-1114. Doi: https://doi.org/10.21577/0103-5053.20190003
115. Xia D, Shi J, Gong J, Wu X, Yang Q, Zang Y. Antioxidant activity of Chinese mei (*Prunus mume*) and its active phytochemicals. J Med Plant Res. 2010;4(12):1156-1160.
116. Bopitiya D, Madhujith T. Antioxidant potential of pomegranate (*Punica granatum* L.) cultivars grown in Sri Lanka. Trop Agric Res. 2015;24(1):71. Doi: https://doi.org/10.4038/tar.v24i1.7990
117. Yi F, Sun J, Bao X, Ma B, Sun M. Influence of molecular distillation on antioxidant and antimicrobial activities of Rose Essential Oils. LWT-Food Sci Technol. 2019. 102:310-316. Doi: https://doi.org/10.1016/j.lwt.2018.12.051

118. Karadağ AE, Demirci B, Çaşkurlu A, Demirci F, Okur ME, Orak D, *et al*. *In vitro* antibacterial, antioxidant, anti-inflammatory and analgesic evaluation of *Rosmarinus officinalis* L. Flower extract fractions. S Afr J Bot. 2019;125:214-220. Doi: https://doi.org/10.1016/j.sajb.2019.07.039
119. Kukrić Z, Vulić J, Jazić M. Polyphenol content and antioxidant activity of wild and cultivated blackberry (*Rubus Fruticosus* L.) juices. J Chem Technol Environ. 2020;1(1):21-27. Doi: https://doi.org/https://doi.org/10.7251/JCTE2001021K
120. Veljković B, Jakovljević V, Stanković M, ajić-Stevanović Z. Phytochemical and antioxidant properties of fresh fruits and some traditional products of wild grown raspberry (*Rubus idaeus* L.). Not Bot Horti Agrabo. 2019;47(3):563-573. Doi: https://doi.org/10.15835/nbha47311465
121. Motham P, Thonpho A, Srihanam P. Phytochemical and antioxidation of fractionated sugercane: suphanburi 50 variety. MSU Editorial Board for Proceeding. 2020;65.
122. Hernández-Pérez T, Valverde ME, Orona-Tamayo D, Paredes-Lopez O. Chia (*Salvia hispanica*): Nutraceutical properties and therapeutic applications. The 2nd International Conference of Ia ValSe-Food Network. 2020. Doi: https://doi.org/10.3390/proceedings2020053017
123. Ruslan K, Happyniar S, Fidrianny I. Antioxidant potential of two varieties of *Sesamum indicum* L. collected from Indonesia. J Taibah Univ Med Sci. 2018;13(3):211-218. Doi: https://doi.org/10.1016/j.jtumed.2018.02.004
124. Magalhães FS, Cassiano DSA, Branco CRC, Silva TRS, Lins ACS, Silva MS, *et al*. Antioxidant activity and phenolics analysis by HPLC-dad of solanum *Thomasiifolium sendtner* (Solanaceae). Free Radicals and Antioxidants. 2014;4(1):15-23. Doi: https://doi.org/10.5530/fra.2014.1.4
125. Kusmiati K, Afiati F, Widhiani C, Aditia A, Elviani DD., Kanti A. The potential of lutein extract of *Tagetes erecta* L. flower as an antioxidant and enhancing phagocytic activity of macrophage cells. Innovation in the Food Sector Through the Valorization of Food and Agro-Food By-Products. 2021, p.179-192.
126. Sasidharan I, Sundaresan A, Nisha VM, Kirishna MS, Raghu KG, Jayamurthy P. Inhibitory effect of *Terminalia chebula*retz. fruit extracts on digestive enzyme related to diabetes and oxidative stress. J Enzyme Inhub Med Chem. 2012;27(4);578-586. Doi: https://doi.org/10.3109/14756366.2011.603130
127. Köksal E, Bursal E, Gülçin İ, Korkmaz M, Çağlayan C, Gören AC, *et al*. Antioxidant activity and polyphenol content of Turkish thyme (*Thymus vulgaris*) monitored by liquid chromatography and Tandem Mass Spectrometry. Int J Food Prop. 2016;20(3):514-525. Doi: https://doi.org/10.1080/10942912.2016.1168438
128. Priya V, Jananie RK, Vijayalakshmi K. Studies on anti-oxidant activity of *Trigonella foenum* graecum seed using *in vitro* models. Int J Pharm Sci Res. 2011;2(10):2704-2708.
129. Abeysekera WK, Jayawardana SA, Abeysekera WP, Yathursan S, Premakumara GA, Ranasinghe P. Antioxidant potential of selected whole grain cereals consumed by Sri Lankans: A comparative *in vitro* study. Sri Lankan J Biol. 2017;2(2):12. Doi: https://doi.org/10.4038/sljb.v2i2.9
130. Kalin P, Gülçin İ, Gören AC. Antioxidant activity and polyphenol content of cranberries (*Vaccinium macrocarpon*). Rec Nat Prod. 2015;9(4):496-502.
131. Zeghad N, Ahmed E, Belkhiri A, Heyden YV, Demeyer K. Antioxidant activity of *Vitis vinifera, Punica granatum, Citrus aurantium* and *Opuntia Ficus* indica fruits cultivated in Algeria. Heliyon. 2019;5(4). Doi: https://doi.org/10.1016/j.heliyon.2019.e01575
132. Basha SA, Rao UJSP. Bioactivities of fractions obtained from Green Gram (*Vigna radiata*) milled by-products. Food Biosci. 2017;19:134-141. Doi: https://doi.org/10.1016/j.fbio.2017.07.004
133. Ali AM, El-Nour MEA, Yagi SM. Total phenolic and flavonoid contents and antioxidant activity of ginger (*Zingiber officinale* Rosc.) rhizome, callus, and callus treated with some elicitors. J Genet Eng & Biotechnol. 2018;16(2):677-682. Doi: https://doi.org/10.1016/j.jgeb.2018.03.003
134. Hwang JS, Cho CH, Baik MY, Park SK, Heo HJ, Cho YS, *et al*. Effects of freeze-drying on antioxidant and anticholinesterase activities in various cultivars of kiwifruit (*Actinidia* spp..). Food Sci Biotechnol. 2017;26(1):221-228. Doi: https://doi.org/10.1007/s10068-017-0030-5

135. Ingkaninan K, Temkitthawon P, Chuenchom K, Yuyaem T, Thongnoi W. Screening for acetylcholinesterase inhibitory activity in plants used in Thai traditional rejuvenating and neurotonic remedies. J Ethnopharmacol. 2003;89(2-3):262-264. Doi: https://doi.org/10.1016/j.jep.2003.08.008
136. Nile A, Nile SH, Kim DH, Keum YS, Seok PG, Sharma K. Valorization of onion solid waste and their flavonols for assessment of cytotoxicity, enzyme inhibitory and antioxidant activities. Food Chem Toxicol. 2018;119:281-289. Doi: https://doi.org/10.1016/j.fct.2018.02.056
137. Suttisansanee U, Thiyajai P, Chalermchaiwat P, Wongwathanarat K, Pruesapan K, Charoenkiatkul S, *et al*. Phytochemicals and *in vitro* bioactivities of aqueous ethanolic extracts from common vegetables in Thai food. Plant. 2021;10(8):1563. Doi: https://doi.org/10.3390/plants10081563
138. Shaker MA. The effect of *Aloe vera* extract on acetylcholinesterase ache and monoamine oxidase MAO enzymes. Al-Mustansiriyah Journal of Science. 2019;29(4):88-92. Doi: https://doi.org/10.23851/mjs.v29i4.411
139. Chaiyana W, Sriyab S, Okonogi S. Enhancement of cholinesterase inhibition of *Alpinia Galanga* (L.) Willd. essential oil by microemulsions. Molecules. 2019;891:14-20. Doi: https://doi.org/10.4028/www.scientific.net/amm.891.14
140. Nanasombat S, Yansodthee K, Jongjaited I. Evaluation of antidiabetic, antioxidant and other phytochemical properties of Thai fruits, vegetables and some local food plants. Walailak J Sci Technol. 2019;16(1):851-866. Doi: https://doi.org/10.48048/wjst.2019.3731
141. Wang Y, Chen X, Zhao C, Miao J, Mao X, Li X, *et al*. Effects of temperature during processing with wine on chemical composition, antioxidant capacity and enzyme inhibition activities of *Angelica sinensis* radix. Int J Food Sci Tech. 2017;52(6):1324-1332. Doi: https://doi.org/10.1111/ijfs.13403
142. Gholamhoseinian A, Moradi MN, Sharifi-far F. Screening the methanol extracts of some Iranian plants for acetylcholinesterase inhibitory activity. Res Pharm Sci. 2009;4(3):105-112.
143. Mathew M, Subramanian S. *In vitro* screening for anti-cholinesterase and antioxidant activity of methanolic extracts of ayurvedic medicinal plants used for cognitive disorders. PLoS ONE. 2014;9(1). Doi: https://doi.org/10.1371/journal.pone.0086804
144. Rehman S, Ali Ashfaq U, Sufyan M, Shahid I, Ijaz B, Hussain M. The insight of *in silico* and *in vitro* evaluation of beta vulgaris phytochemicals against Alzheimer's disease targeting acetylcholinesterase. PLoS ONE. 2022;17(3). Doi: https://doi.org/10.1371/journal.pone.0264074
145. Abdelwahab SI. *In vitro* inhibitory effect of Boeserngin A on human acetylcholinerase: understanding its potential using *in silico* ADMET studies. J Appl Pharm Sci. 2013;3(3):30-35. Doi: https://doi.org/10.7324/japs.2013.30306
146. Ercetin T, Senol FS, Erdogan OI, Toker G. Comparative assessment of antioxidant and cholinesterase inhibitory properties of the marigold extracts from *Calendula arvensis* L. and *Calendula officinalis* L. Ind Crops Prod. 2012;36(1):203-208. Doi: https://doi.org/10.1016/j.indcrop.2011.09.007
147. Suttisansanee U, Kunkeaw T, Thatsanasuwan N, Tonglim J, Temviriyanukul P. The investigation on cholinesterases and BACE1 inhibitory activities in various tea infusions. Walailak J Sci Technol. 2019;16(3):165-174. Doi: https://doi.org/10.48048/wjst.2019.6221
148. Loizzo MR, Tundis R, Menichini F, Statti GA, Menichini F. Influence of ripening stage on health benefits properties of *Capsicum annuum* var. acuminatum L.: *in Vitro* studies. J Med Food. 2008;11(1):189-189. Doi: https://doi.org/10.1089/jmf.2007.638
149. Nantakornsuttanan N, Thuphairo K, Kukreja RK, Charoenkiatkul S, Suttisansanee U. Anti-cholinesterase inhibitory activities of different varieties of chili peppers extracts. Int Food Res J. 2016;23(5):1953-1959.
150. Lim SS, Han SM, Kim SY, Bae YS, Kang IJ. Isolation of Acetylcholinesterase Inhibitors from the Flowers of *Chrysanthemum indicum* Linne. Food Sci Biotechnol. 2007;16(2):265-269.
151. Jazayeri SB, Amanlou A, Ghanadian N, Pasalar P, Amanlou M. A preliminary investigation of anticholinesterase activity of some Iranian medicinal plants commonly used in traditional medicine. DARU J Pharm Sci. 2014;22:1-5.

152. Tundis R, Loizzo MR, Bonesi M, Menichini F, Mastellone V, Colica C, *et al*. Comparative study on the antioxidant capacity and cholinesterase inhibitory activity of *Citrus aurantifolia* swingle, *C. aurantium* L., and *C. Bergamia* Risso and Poit. peel essential oils. J Food Sci. 2012;77(1). Doi: https://doi.org/10.1111/j.1750-3841.2011.02511.x
153. Aazza S, Lyoussi B, Miguel MG. Antioxidant and antiacetylcholinesterase activities of some commercial essential oils and their major compounds. Molecules. 2011;16(9):7672-7690. Doi: https://doi.org/10.3390/molecules16097672
154. Ademosun AO, Oboh G. Inhibition of acetylcholinesterase activity and fe^{2+}-induced lipid peroxidation in rat brain *in vitro* by some citrus fruit juices. J Med Food. 2012;15(5):428-434. Doi: https://doi.org/10.1089/jmf.2011.0226
155. Shen X, Nie F, Fang H, Liu K, Li Z, Li X, *et al*. Comparison of chemical compositions, antioxidant activities, and acetylcholinesterase inhibitory activities between coffee flowers and leaves as potential novel foods. Food Sci Nut. 2022;11(2):917-929. Doi: https://doi.org/10.1002/fsn3.3126
156. Shwetha V, Veena SM, Govindappa M, Zameer F, Francois NN, More SS. *In vitro* neutralization of *Naja naja*venom enzymes by folk medicinal plant extracts. J Biol Active Prod Nature. 2019;9(4):278-288. Doi: https://doi.org/10.1080/22311866.2019.1676166
157. Abbasi MA, Ilyas M, Aziz-ur-Rehman SA, Shahwar D, Raza MA, Khan KM, *et al*. Curcumin and its derivatives: Moderate inhibitors of acetylcholinesterase, butyrylcholinesterase and Trypsin. Sci Iran. 2012;19(6):1580-1583. Doi: https://doi.org/10.1016/j.scient.2012.10.014
158. Turkiewicz I, Wojdyło A, Tkacz K, Nowicka P, Hernández F. Antidiabetic, anticholinesterase and antioxidant activity vs. terpenoids and phenolic compounds in selected new cultivars and hybrids of artichoke *Cynara Scolymus* L. Molecules. 2019;24(7):1222. Doi: https://doi.org/10.3390/molecules24071222
159. Yusuf E, Wojdyło A, Oszmiański J, Nowicka P. Nutritional, phytochemical characteristics and *in vitro* effect on α-amylase, α-glucosidase, lipase, and cholinesterase activities of 12 coloured carrot varieties. Foods. 2021;10(4):808. Doi: https://doi.org/10.3390/foods10040808
160. Mahnashi MH, Alshehri OM. Isolation, *in vitro* and *in silico* anti-alzheimer and anti-inflammatory studies on phytosteroids from aerial parts of Fragaria × Ananassa Duch. Biomolecules. 2022;12(10):1430. Doi: https://doi.org/10.3390/biom12101430
161. Cör D, Botić T, Gregori A, Pohleven F, Knez Ž. The effects of different solvents on bioactive metabolites and "*in vitro*" antioxidant and anti-acetylcholinesterase activity of Ganoderma lucidum fruiting body and primordia extracts. Maced J Chem Chem Eng. 2017;36(1): Doi: https://doi.org/10.20450/mjcce.2017.1054
162. Tan WN, Khairuddean M, Wong KC, Khaw KY, Vikneswaran M. New cholinesterase inhibitors from *Garcinia atroviridis*. Fitoterapia. 2014;97:261-267. Doi: https://doi.org/10.1016/j.fitote.2014.06.003
163. Wang Z, Wang W, Zhu C, Gao X, Chu W. Evaluation of antioxidative and neuroprotective activities of total flavonoids from sea buckthorn (*Hippophae rhamnoides* L.). Front Nut. 2022;9. Doi: https://doi.org/10.3389/fnut.2022.861097
164. Kobus-Cisowska J, Szulc P, Szczepaniak O, Dziedziński M, Szymanowska D, Szymandera-Buszka K, *et al*. Variability of *Hordeum vulgare* L. cultivars in yield, antioxidant potential, and cholinesterase inhibitory activity. Sustainability. 2020;12(5):1938. Doi: https://doi.org/10.3390/su12051938
165. Huh E, Kim HG, Park H, Kang MS, Lee B, Oh MS. *Houttuynia cordata* improves cognitive deficits in cholinergic dysfunction Alzheimer's disease-like models. Biomol Ther. 2014;22(3):176-183. Doi: https://doi.org/10.4062/biomolther.2014.040
166. Bhadra S, Mukherjee PK, Kumar NS, Bandyopadhyay A. Anticholinesterase activity of standardized extract of *Illicium verum* hook. F. Fruits. Fitoterapia. 2011;82(3):342-346. Doi: https://doi.org/10.1016/j.fitote.2010.11.003
167. Dhanasekaran S, Perumal P, Palayan M. *In-vitro* screening for acetylcholinesterase enzyme inhibition potential and antioxidant activity of extracts of *Ipomoea Aquatica* Forsk: Therapeutic lead for Alzheimer's disease. J Appl Pharm Sci. 2015;12-16. Doi: https://doi.org/10.7324/japs.2015.50203

168. Begum T, Gogoi R, Sarma N, Pandey SK, Lal M. Direct sunlight and partial shading alter the quality, quantity, biochemical activities of *Kaempferia parviflora* wall., ex Baker rhizome essential oil: A high industrially important species. Ind Crops Prod. 2022;180:114765. Doi: https://doi.org/10.1016/j.indcrop.2022.114765
169. Mocan A, Moldovan C, Zengin G, Bender O, Locatelli M, Simirgiotis M, et al. UHPLC-QTOF-MS analysis of bioactive constituents from two Romanian goji (*lycium barbarum* L.) berries cultivars and their antioxidant, enzyme inhibitory, and real-time cytotoxicological evaluation. Food Chem Toxicol. 2018;115:414-424. Doi: https://doi.org/10.1016/j.fct.2018.01.054
170. Oboh G, Bakare OO, Ademosun AO, Akinyemi, A. J., & Olasehinde, T. A. (2015). Inhibition of Cholinesterases and Some Pro-Oxidant induced Oxidative Stress in Rats Brain by Two Tomato (*Lycopersicon Esculentum*) Varieties. Int J Biomed Sci. 2015;11(1):48-53.
171. Başı Z, Türkoğlu, V, Güler M, Kıvanç MR. *In vitro* Effects of Certain Plant Extracts on Acetylcholinesterase (EC 3.1.1.7) Enzyme in Lake Van Fish Liver and Brain. Hacettepe Journal of Biology and Chemistry. 2016;44(4):435-440. Doi: https://doi.org/10.15671/HJBC.2016.124
172. Eruygur N, Dincel B, Kutuk Dincel NG, Ucar E. Comparative study of *in vitro* antioxidant, acetylcholinesterase and butyrylcholinesterase activity of alfalfa (*Medicago sativa* L.) collected during different growth stages. Open Chem. 2018;16(1):963-967. Doi: https://doi.org/10.1515/chem-2018-0088
173. Dastmalchi K, Ollilainen V, Lackman P, Gennäs GB, Dorman HJD, Järvinen PP, et al. Acetylcholinesterase inhibitory guided fractionation of *Melissa officinalis* L. Bioorg Med Chem. 2009;17(2):867-871. Doi: https://doi.org/10.1016/j.bmc.2008.11.034
174. Ali-Shtayeh MS, Jamous RM, Abu-Zaitoun SY, Khasati AI, Kalbouneh SR. Biological properties and bioactive components of *Mentha spicata* L. essential oil: Focus on potential benefits in the treatment of obesity, Alzheimer's disease, dermatophytosis, and drug-resistant infections. Evid Based Complement Alternat Med. 2019;2019. Doi: https://doi.org/10.1155/2019/3834265
175. Nwidu L, Elmorsy E, Aprioku J, Siminialayi I, Carter W. *In vitro* anti-cholinesterase and antioxidant activity of extracts of *Moringa oleifera* plants from Rivers State, Niger Delta, Nigeria. Medicines. 2018;5(3):71. Doi: https://doi.org/10.3390/medicines5030071
176. Ramirez DA, Carazzone C. Small molecules putative structure elucidation in endemic Colombian fruits: CFM-ID approach. Int J Food Prop. 2022;25(1):2604-2616. Doi: https://doi.org/10.1080/10942912.2022.2147539
177. Singh V, Kahol A, Singh IP, Saraf I, Shri R. Evaluation of anti-amnesic effect of extracts of selected *Ocimum* species using *in-vitro* and *in-vivo* models. J Ethnopharmacol. 2016;193:490-499. Doi: https://doi.org/10.1016/j.jep.2016.10.026
178. Ressaissi A, Attia N, Falé PLV, Pacheco R, Teixeira VH, Machuqueiro M, *et al*. Aqueous Extracts from Nopal (*Opuntia ficus-indica*): Antiacetylcholinesterase and Antioxidant Activity from Phenolic Bioactive Compounds. International Journal of Green and Herbal Chemistry. 2016;5(3):337-348.
179. de Torre MP, Cavero RY, Calvo MI. Anticholinesterase activity of selected medicinal plants from Navarra region of Spain and a detailed phytochemical investigation of *Origanum vulgare* L. Ssp. vulgare. Molecules. 2022;27(20):7100. Doi: https://doi.org/10.3390/molecules27207100
180. Adsersen A, Gauguin B, Gudiksen L, Jäger AK. Screening of plants used in Danish folk medicine to treat memory dysfunction for acetylcholinesterase inhibitory activity. J Ethnopharmacol. 2006;104(3):418-422. Doi: https://doi.org/10.1016/j.jep.2005.09.032
181. Werawattanachai N, Kaewamatawong R. Screening for Acetylcholinesterase Inhibitory Activityfrom the Piperaceae. J Sci Tech UBU. 2019;18(3):26-33.
182. Mejri F, Ben KH, Njim L, Baati T, Selmi S, Martins A, et al. *In vitro* and *in vivo* biological properties of pea pods (*Pisum sativum* L.). Food Biosci. 2019;32:100482. Doi: https://doi.org/10.1016/j.fbio.2019.100482

183. Vahedi-Mazdabadi Y, Karimpour-Razkenari E, Akbarzadeh T, Lotfian H, Toushih M, Roshanravan N, *et al*. Anti-cholinesterase and Neuroprotective Activities of Sweet and Bitter Apricot Kernels (*Prunus armeniaca* L.). Iran J Pharm Res. 2020;19(4):216-224. Doi: https://doi.org/ 10.22037/ijpr.2019.15514.13139
184. Rybak M, Wojdyło A. Inhibition of α-amylase, α-glucosidase, pancreatic lipase, 15-lipooxygenase and acetylcholinesterase modulated by polyphenolic compounds, organic acids, and carbohydrates of *Prunus domestica* fruit. Antioxidants. 2023;12(7):1380. Doi: https://doi.org/10.3390/antiox12071380
185. Akyüz M. The determination of antidiabetic, anticholinesterase and antioxidant properties of ethanol and water extracts of blackberry (*Rubus fruticosus* L.) fruits at different maturity stages. S AfrJ Bot. 2022;151:1035-1048. Doi: https://doi.org/10.1016/j.sajb.2022.11.012
186. Kocakaya SO, Ertas A, Yener I, Ercan B, Oral EV, Akdeniz M, *et al*. Selective *in-vitro* Enzymes' Inhibitory Activities of Fingerprints Compounds of Salvia Species and Molecular Docking Simulations. Iran J Pharm Res. 2020;19(2):187-198. Doi: https://doi.org/10.22037/ijpr.2020.112498.13801
187. Kim MY, Kim S, Lee J, Kim JI, Oh E, Kim SW, *et al*. Lignan-rich sesame (*Sesamum indicum* L.) cultivar exhibits *in vitro* anti-cholinesterase activity, anti-neurotoxicity in amyloid-β induced SH-SY5Y cells, and produces an in vivo nootropic effect in scopolamine-induced memory impaired mice. Antioxidants. 2023;12(5):1110. Doi: https://doi.org/10.3390/antiox12051110
188. Senizza B, Rocchetti G, Sinan KI, Zengin G, Mahomoodally MF, Glamocilja J, et al. The phenolic and alkaloid profiles of *Solanum erianthum* and *Solanum torvum* modulated their biological properties. Food Biosci. 2021;41:100974. Doi: https://doi.org/10.1016/j.fbio.2021.100974
189. Moliner C, Barros L, Dias M, López V, Langa E, Ferreira I, *et al*. Edible flowers of *Tagetes erecta* L. as functional ingredients: Phenolic composition, antioxidant and protective effects on *Caenorhabditis elegans*. Nutrients. 2018;10(12):2002. Doi: https://doi.org/10.3390/nu10122002
190. Tkacz K, Wojdyło A, Nowicka P, Turkiewicz I, Golis T. Characterization *in vitro* potency of biological active fractions of seeds, skins and flesh from selected Vitis vinifera l. cultivars and interspecific hybrids. J Funct Foods. 2019;56:353-363. Doi: https://doi.org/10.1016/j.jff.2019.03.029
